# Virulence and Antibiotic Resistance of aEPEC/STEC *Escherichia coli* Pathotypes with Serotype Links to *Shigella boydii* 16 Isolated from Irrigation Water

**DOI:** 10.3390/pathogens14060549

**Published:** 2025-06-01

**Authors:** Yessica Enciso-Martínez, Edwin Barrios-Villa, Manuel G. Ballesteros-Monrreal, Armando Navarro-Ocaña, Dora Valencia, Gustavo A. González-Aguilar, Miguel A. Martínez-Téllez, Julián Javier Palomares-Navarro, Fernando Ayala-Zavala

**Affiliations:** 1 Departamento de Ciencias Químico-Biológicas y Agropecuarias, Universidad de Sonora, Caborca 83621, Mexico; yessica.enciso@unison.mx (Y.E.-M.); edwin.barrios@unison.mx (E.B.-V.); manuel.ballesteros@unison.mx (M.G.B.-M.); dora.valencia@unison.mx (D.V.); 2 Centro de Investigación en Alimentación y Desarrollo, A.C., Carretera Gustavo Enrique Astiazarán Rosas 46, Col. La Victoria, Hermosillo 83304, Mexico; gustavo@ciad.mx (G.A.G.-A.); julian.palomares.220@estudiantes.ciad.mx (J.J.P.-N.); norawa@ciad.mx (M.A.M.-T.); 3 Departamento de Salud Pública, Facultad de Medicina, Universidad Nacional Autónoma de México, Ciudad Universitaria, Ciudad de México 04510, Mexico; arnava@unam.mx

**Keywords:** hetero-pathogenic, pathotypes, serotype cross-reactivity, antibiotic-resistance, virulence, food safety

## Abstract

Irrigation water can serve as a reservoir and transmission route for pathogenic *Escherichia coli*, posing a threat to food safety and public health. This study builds upon a previous survey conducted in Hermosillo, Sonora (Mexico), where 445 samples were collected from a local Honeydew melon farm and associated packing facilities. Among the 32 *E. coli* strains recovered, two strains, A34 and A51, were isolated from irrigation water and selected for further molecular characterization by PCR, due to their high pathogenic potential. Both strains were identified as hybrid aEPEC/STEC pathotypes carrying *bfpA* and *stx1* virulence genes. Adhesion assays in HeLa cells revealed aggregative and diffuse patterns, suggesting enhanced colonization capacity. Phylogenetic analysis classified A34 within group B2 as associated with extraintestinal pathogenicity and antimicrobial resistance, while A51 was unassigned to any known phylogroup. Serotyping revealed somatic antigens shared with *Shigella boydii* 16, suggesting possible horizontal gene transfer or antigenic convergence. Antibiotic susceptibility testing showed resistance to multiple β-lactam antibiotics, including cephalosporins, linked to the presence of *bla_CTX-M-151_* and *bla*_CTX-M-9_. Although no plasmid-mediated quinolone resistance genes were detected, resistance may involve efflux pumps or mutations in *gyrA* and *parC*. These findings are consistent with previous reports of *E. coli* adaptability in agricultural environments, suggesting potential genetic adaptability. While our data support the presence of virulence and resistance markers, further studies would be required to demonstrate mechanisms such as horizontal gene transfer or adaptive evolution.

## 1. Introduction

Water used for irrigation is a critical component of global agricultural systems, essential for crop development and food production. However, its microbiological quality has direct implications for food safety, public health, and environmental sustainability [1]. Using contaminated water in primary production is considered one of the major sources of fresh produce contamination [1]. Waste from dairy farms, including fecal matter, manure slurry, and wastewater, has been increasingly recognized as a significant environmental reservoir of antimicrobial-resistant and pathogenic *E. coli*. These residues often carry a high load of antibiotic-resistance genes and virulence-associated genes, which can disseminate through runoff or irrigation practices, contributing to the microbiological contamination of surface water sources used in agriculture [2,3]. The World Health Organization (WHO) and Food and Agriculture Organization (FAO) have reported that microbial contamination in fresh produce contributes to a significant proportion of foodborne illness outbreaks globally, with *Escherichia coli* being among the most frequent etiological agents [4]. In Latin America, approximately 70% of surface water used for irrigation in agricultural zones is untreated, increasing the likelihood of fecal contamination [5]. In this context, *E. coli* not only serves as a fecal indicator organism, but specific pathogenic variants, including diarrheagenic *E. coli* (DEC), pose a direct health risk due to their ability to cause gastroenteritis and severe systemic infections [6]. Among these, Enteropathogenic *E. coli* (EPEC) and Shiga-toxigenic *E. coli* (STEC) are especially relevant. EPEC strains are subclassified as typical (tEPEC), which carry the *bfpA* gene encoding the bundle-forming pilus, and atypical (aEPEC), which lack this gene [7]. On the other hand, STEC strains are responsible for severe diseases such as hemorrhagic colitis and hemolytic uremic syndrome. They are characterized by *stx1* and/or *stx2* genes, which encode Shiga toxins [8].

Moreover, the emergence of hetero-pathogenic or hybrid *E. coli* strains that combine virulence traits of different pathotypes (e.g., aEPEC/STEC) or of both DEC and ExPEC (extraintestinal pathogenic *E. coli*) represents a growing concern. These strains often display increased pathogenicity and resistance, complicating diagnosis and treatment [9]. In addition to pathotyping, serotyping remains a valuable tool in epidemiological surveillance. For example, WHO identified 12 serogroups classically associated with EPEC in 1987 [10], while serogroups such as O157, O26, and O111 are commonly linked to STEC. Recent reports have described *E. coli* strains sharing somatic antigens with *Shigella boydii* 16, including those isolated from pediatric patients with diarrhea and from animals raised for human consumption, suggesting horizontal gene transfer and a broader ecological niche [11].

Despite these advances, several knowledge gaps remain. Most surveillance studies focus on clinical or food isolates, with limited data on *E. coli* strains circulating in agricultural systems. Although relatively few studies have characterized environmental *E. coli* strains at both the genotypic and phenotypic levels, particularly regarding their hybrid virulence profiles and antimicrobial resistance, recent research has begun to address this gap. Despite these advances, several knowledge gaps remain. Most surveillance studies focus on clinical or food isolates, with limited data on *E. coli* strains circulating in agricultural systems. Although relatively few studies have characterized environmental *E. coli* strains at both the genotypic and phenotypic levels, particularly regarding hybrid virulence profiles and antimicrobial resistance [12], recent research has begun to address this gap. For instance, identified multidrug-resistant *E. coli* strains in the dairy farm environment carry diverse virulence-associated genes and antibiotic resistance determinants [2,13]. Similarly, whole-genome sequencing of ESBL-producing *E. coli* isolates from environmental sources revealed the co-occurrence of *bla*_OXA−1_, *catB3*, and *arr-3* genes, along with transferable resistance elements and phylogenetic diversity [3]. This emerging body of evidence highlights the complexity of environmental *E. coli* and underscores the need for integrated surveillance approaches. This gap is significant given the role of irrigation water as a contamination route in fresh produce supply chains. This study builds upon previous work in Hermosillo, Sonora, Mexico [14], where 445 environmental samples were collected from a Honeydew melon farm and its packing facilities, leading to the isolation of 32 *E. coli* strains. Among these, strains A34 and A51, isolated from irrigation water, stood out due to their virulence and resistance profiles. These isolates were identified as aEPEC/STEC pathotypes and displayed serotype links to *S. boydii* 16, raising important questions regarding their origin, evolution, and potential public health risk. We hypothesize that the *E. coli* strains A34 and A51 may represent emerging hybrid pathotypes. The coexistence of virulence and resistance genes suggests the possible influence of environmental pressures and horizontal gene transfer, although the mechanisms were not directly investigated in this study. Based on their genetic makeup, adhesion phenotypes, and resistance to β-lactam antibiotics, these strains exemplify the evolutionary dynamics of *E. coli* in contaminated agricultural environments. The present study aims to address these gaps by providing a detailed molecular and phenotypic characterization of strains A34 and A51, thereby contributing to a better understanding of the microbial risks associated with irrigation water.

## 2. Materials and Methods

### 2.1. Bacterial Strains

This study builds upon a previous investigation conducted in Hermosillo, Sonora, Mexico, which focused on understanding the environmental reservoirs of pathogenic *E. coli* [14]. In that investigation, 445 environmental samples were collected from a Honeydew melon production system, including irrigation water, soil, packing materials, workers’ hands, and fruit surfaces. A total of 32 *E. coli* strains were isolated, of which 59% originated from irrigation water, highlighting its role as a primary contamination route in agricultural environments. Among these isolates, two strains, A34 and A51, were selected for detailed analysis due to their rare serological profile, specifically their somatic antigenic similarity with *S. boydii* 16, as determined by agglutination assays. This unusual finding suggests possible horizontal gene transfer or antigenic convergence between *E. coli* and *Shigella* species, a phenomenon that is infrequently reported in environmental isolates. Because of their unique serotype identity, these strains were particularly interesting for further characterization.

### 2.2. Isolation and Biochemical Characterization of E. coli

The samples were inoculated on MacConkey and Eosin Methylene Blue (Becton, Dickson and Company, Sparks, Baltimore, MD, USA) and incubated for 18 h at 37 ± 2 °C. Following that, biochemical characterization was confirmed through a series of standard biochemical assays, which included mobility, indole, sulfhydric acid production, glucose fermentation, lactose fermentation, Simmons citrate, Voges-Proskauer, urea, gas production, methyl red, and lysine decarboxylase [15].

### 2.3. Genomic DNA Extraction

The *E. coli* strains displaying the biochemical characteristics were processed for DNA extraction using the alkaline lysis method following the established protocols in The Molecular Cloning Laboratory Manual 2012 [16]. The extracted DNA was preserved at −20 °C for preservation.

### 2.4. Molecular Identification of E. coli

The *E. coli* strains were identified using a conventional PCR with GoTaq Green Master Mix (Promega, Madison, WI, USA) targeting the *ybbW* (Table S1), which encodes the allantoin transporter protein and is exclusive to this species [17,18]. The amplified PCR product was visualized via electrophoresis on a 1% agarose gel in 1× TAE buffer with GelStarTM Stain (Lonza, Morristown, NJ, USA). After confirmation of the identification, the *E. coli* strains were maintained in Luria-Bertani broth (Becton, Dickson and Company, Sparks, MD, USA) containing 20% *v*/*v* glycerol and stored at −80 °C [19].

### 2.5. Determination of Diarrheagenic Pathotypes

Conventional PCR was performed to search for genetic pathotype markers: the *eaeA* gene for EPEC/EHEC, the *bfpA* gene for EPEC, the *stx1* and *stx2* genes for EHEC/STEC, thermolabile (LT) and thermostable (ST) toxins for ETEC, the *daaE* and *afa/draBC* genes for DAEC, the *ial* for EIEC, and the pCVD for EAEC identification using specific primers (Table S1).

### 2.6. Adherence Assay

HeLa cells were cultured in six-well polystyrene plates containing sterile glass coverslips, using Dulbecco’s Modified Eagle Medium (DMEM) supplemented with 5% fetal bovine serum (FBS), and incubated at 37 °C in a 5% CO_2_ atmosphere until reaching approximately 80% confluence. A cell suspension of 5 × 10⁴ cells/mL was then prepared in 2 mL of antibiotic-free DMEM supplemented with 10% FBS and incubated overnight under the same conditions.

After incubation, HeLa monolayers were gently washed with sterile phosphate-buffered saline (PBS), and 2 mL of fresh DMEM with 10% FBS was added to each well. A 0.5 McFarland suspension of each *E. coli* isolate, prepared from an 18–24 h preculture in brain heart infusion broth, was diluted in DMEM and applied to the HeLa cells at a multiplicity of infection (MOI) of 30:1 (bacteria: cell). The co-cultures were incubated for 3 h at 37 °C with 5% CO_2_.

Following incubation, the monolayers were washed with PBS to remove non-adherent bacteria, fixed with methanol, stained with Giemsa, and washed three times with PBS. Coverslips were removed and mounted on microscope slides for observation under light microscopy at 1000× magnification. *E. coli* strain K-12 (commensal) was a negative adherence control. Each isolate was evaluated in three independent assays, and adherence patterns were classified qualitatively as aggregative, diffuse, or localized, based on previously described morphological criteria [19].

### 2.7. Serotyping

To establish a serotype-based classification of the strains, agglutination assays were conducted utilizing 96-well microtitre plates, and rabbit serum (SERUNAM) was obtained a2.1 Bacterial strains against 186 somatic antigens and 53 flagellar antigens for *E. coli*, as well as 45 somatic antigens for *Shigella* species, following the methods described by Ørskov and Ørskov [20].

### 2.8. Phylogenetic Group Determination

The phylogenetic group of the identified strains was determined using the scheme previously proposed by Clermont in 2013 [21] (Table S1).

### 2.9. Virulence-Associated Genes

Eleven virulence genes associated with 9 virulence factors were evaluated: cytotoxic necrotizing factor (*cnf-1*), secreted autotransporter toxin (*sat*), type II capsule synthesis (*kpsMII*), catecholate siderophore receptor (*iroN*), adhesins (*afa* and *afa/draBC*), type P pilus (*papGII* and *papGIII*), cerebral endothelial invasion (*ibeA*), hemolysin A (*hlyA*) and total distension toxin (*cdtB*). Conventional PCR was used using GoTaq Green MasterMix (Promega) and specific primers for each gene (Table S1). The PCR products were resolved on a 1% agarose gel [19]. CFT073 and J96 were used as positive controls.

### 2.10. Enterobacterial Repetitive Intergenic Consensus-Polymerase Chain Reaction (ERIC-PCR)

To establish a phylogenetic relationship, an ERIC-PCR was performed using previously reported primers (Table S1) [22]. The reaction tube contained 0.4 μM of each primer, GoTaq MasterMix (Promega Corporation, Madison WI, USA) (containing reaction buffer, 400 μM of each dNTP, 3 mM MgCl_2_, and 1× Taq^®^ DNA polymerase) to a final volume of 12 μL in a MiniAmp Plus thermal cycler (Applied BioSystems, Foster City, CA, USA). The protocol includes a denaturation cycle at 95 °C followed by 35 denaturation cycles at 95 °C for 60 s, 50 °C for 60 s, and 72 °C for eight minutes, and a final extension cycle for 16 min. The amplicons were resolved in 1% agarose gel electrophoresis and ethidium bromide to visualize them using a UVP transilluminator (AnalytikJena, Beverly, MA, USA). The distance matrices were first obtained with Phyelph 1.4 by placing the bands in each gel lane. A 5% distance between bands was considered part of the same cluster for the clustering, and the UPGMA algorithm and the Dice coefficient were used. A csv file of the distance matrices was then generated with PhyloM to make the dendrogram in MEGA 11.

### 2.11. Antibiotic Susceptibility

The Kirby-Bauer disc diffusion method was used to conduct the antibiogram, in which the commonly prescribed antibiotics for *E. coli* infections were tested. These included amikacin (30 µg), ampicillin (10 µg), aztreonam (30 µg), cefotaxime (31 µg), cefuroxime (30 µg), ceftriaxone (32 µg), cefepime (30 µg), ciprofloxacin (5 µg), ertapenem (10 µg), amoxicillin/clavulanic acid (20/10 µg), meropenem (10 µg), and sulfamethoxazole/trimethoprim (1.25/23.75 µg). The *E. coli* strains of our study and *E. coli* ATCC 25922 (control) were cultured in 5 mL Mueller Hinton broth and incubated at 35 °C ± 2 °C for 16–18 h. The diameters of the inhibition zones were measured in millimeters, and interpretation of susceptibility (susceptible, intermediate, or resistant) was performed according to the Clinical and Laboratory Standard Institute (CLSI) [23], using the standardized zone diameter breakpoints provided for each antimicrobial tested. Furthermore, Magiorakos’ criteria were followed to categorize the *E. coli* strains as non-multidrug-resistant (NMDR), multidrug-resistant (MDR), and extremely drug-resistant (XDR).

### 2.12. Molecular Identification of Extended Spectrum β-Lactamases (ESBLs) and Non-ESBL Genes

The *E. coli* strains were examined for the presence of antibiotic-resistance genes using conventional PCR. Specific primers for ESBL genes (*bla*_CTX-M-1_, *bla*_CTX-M-2_, *bla*_CTX-M-9_, *bla*_CTX-M-151_, *bla*_TEM_, *bla*_SHV_) and non-ESBL genes (*qepA* and *aac(6′)-lb-cr*) were used and listed in Table S1. Each PCR reaction contained 12.5 µL GoTaq Green Master Mix (Promega Corporation, Madison, WI, USA), 0.5 µL of each primer (10 µM), 1.5 µL of template DNA (50–75 ng), and nuclease-free water to make a final volume of 25 µL. The PCR product was then examined through electrophoresis on a 1% agarose gel.

## 3. Results and Discussion

### 3.1. Diarrheagenic Pathotypes

**Figure 1:**
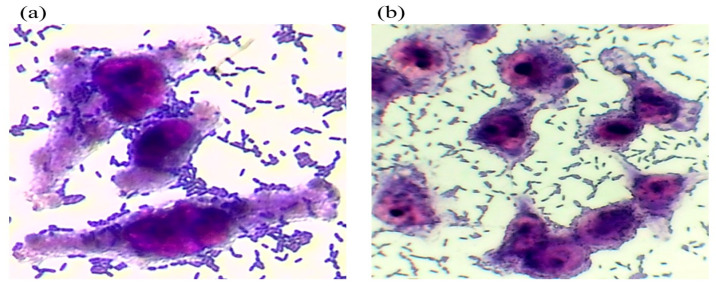
Pattern of aggregative and diffuse adhesion in *E. coli* strain A34 (**a**) and A51 (**b**).

A previous study conducted in Hermosillo, Sonora [14] examined 445 samples obtained from a local farm and packing facilities of Honeydew melon. The results revealed the presence of 32 *E. coli* strains. Among these, two specific strains, A34 and A51, were identified, isolated from irrigation water, and characterized by their high pathogenicity. These strains contained the *bfpA* and *stx1* genes, suggesting they belong to the aEPEC/STEC pathotype (Table 1). To better understand the adherence behavior of the isolates, assays were performed on HeLa cell monolayers. Strains A34 and A51 exhibited distinct adherence patterns, classified qualitatively as aggregative and diffuse (Figure 1). Specifically, Figure 1a shows a compact and dense clustering of bacterial cells on the surface of HeLa, indicative of a strong aggregative adherence pattern. In contrast, Figure 1b illustrates a more scattered distribution of bacteria across the cell monolayer, consistent with a diffuse adherence pattern, although bacterial attachment remains evident. The presence of *bfpA* and *stx1* genes suggests that these strains may possess virulence potential associated with aEPEC and STEC pathotypes. However, functional expression of these genes was not assessed, and the actual impact on pathogenicity remains to be determined. The observed aggregative and diffuse adherence patterns in HeLa cells are consistent with previously described phenotypes, but further studies using host-relevant models (e.g., intestinal organoids or animal infections) are necessary to confirm their role in disease.

The hetero-pathogens aEPEC/STEC of the *E. coli* strains A34 and A51 show combinations of virulence factors. These hetero-pathogens are strictly enteropathogenic, identified by diarrheagenic *E. coli* pathotypes associated with specific virulence factors [8]. Detecting hetero-pathogenic *E. coli* strains in various contexts, including the environment and animals but predominantly in human infections, highlights their relevance. For example, a study in Limpopo, South Africa, showed that 25.3% of children hospitalized for diarrhea had the EAEC/ETEC pathotype [24]. Similarly, in Sweden, the STEC/ETEC pathotype incidence in a clinical collection was 2.05% [25]. Therefore, the identification of EAEC/ETEC and STEC/ETEC pathotypes highlights the dynamic evolution of virulence in *E. coli*, raising concerns about their potential to cause more severe or persistent infections. However, the actual impact of combined pathogenic mechanisms in these strains requires further investigation.

Environmental water is a significant vector for these strains. In Johannesburg, South Africa, several hetero-pathogenic strains were identified, including EHEC/ETEC (1.8%), EAEC/aEPEC (7.6%), EPEC/ETEC (2.4%), EAEC/ETEC (3%), and EPEC/EHEC (1.8%) [26]. In the animal context, hetero-pathogenic pathotypes have been documented in pigs, as indicated by a study in Spain, where 8.1% of ETEC/STEC was identified [27]. Additionally, in Mexico, 4.5% of STEC/ETEC was observed in wild animals (deer), suggesting that these animals can be reservoirs of pathogenic *E. coli* strains [28], raising the possibility of transfer of these strains to the environment, including irrigation water. Runoff from feces, combined with inadequate water management practices, increases the probability of pathogen transfer to crops, potentially leading to foodborne illnesses [29]. This highlights the potential for these pathotypes to spread beyond animal reservoirs, potentially contaminating environmental water sources and posing a significant risk to both public health and agricultural practices.

Various microbiological quality control strategies have been developed and implemented to mitigate the risk of contamination by heteropathogenic *E. coli* strains in irrigation water. These strategies include membrane filtration, chlorine dioxide treatment, ultraviolet radiation, ozone disinfection, and sodium hypochlorite chlorination [30]. Applying these preventive measures is crucial to ensure the safety of agricultural products intended for human consumption. Good Agricultural Practices (GAP) are also essential in preventing pathogenic contamination in food production. GAPs related to irrigation water management, such as risk assessments, implementing appropriate irrigation systems, and periodic microbiological testing of agricultural water, are vital to reducing the risk of pathogen transmission through crops [31]. Using *E. coli* to indicate fecal contamination and the potential presence of human enteric pathogens in irrigation water effectively manages food safety [32]. Since *E. coli* can persist in the environment for extended periods, its detection and monitoring provide valuable insights into the microbiological quality of water used in agriculture, helping prevent foodborne illnesses. Over the years, these strategies have proven effective to some extent; however, challenges remain in terms of sensitivity, cost, and speed, particularly in large-scale agricultural settings. Recent advancements, such as rapid on-site diagnostic tools and real-time monitoring systems, hold promise for improving detection accuracy and reducing delays in identifying contamination. In the future, the integration of these innovative technologies with environmental data analysis could lead to more effective and proactive strategies for managing water quality, ultimately reducing the risk of foodborne diseases and ensuring safer agricultural practices.

### 3.2. Serotyping

During the serotyping of *E. coli* strains from irrigation water, it was found that strain A34 belonged to serogroup H7: O **, while strain A51 belonged to H-:O ** (Table 1). Both strains shared an identity with the somatic antigen of *S. boydii* 16, as demonstrated by serological tests (Table 2). However, unlike *S. boydii* 16, which is immobile due to a deletion in the *flhDC* flagellar operon [33], *E. coli* strains A34 and A51 were mobile. Only strain A34 exhibited the specific flagellar antigen (H7), detected through serological assays. The H7 flagellar antigen plays a crucial role in the colonization and pathogenicity of the strains in the host. This antigen, related to flagellin, triggers an immune response by stimulating the secretion of pro-inflammatory chemokines in human intestinal cells. The expression of flagellar genes is regulated in response to specific environmental signals, inhibiting flagellar biosynthesis to conserve energy and minimize detection by the host immune system [34].

Furthermore, it was discovered that the somatic antigen of *E. coli* strains A34 and A51 was identical to that of *S. boydii* 16. This phenomenon can be explained by the presence of common or closely related somatic antigens between *E. coli* and *Shigella* serotypes, which has been documented. For example, strains of *S. boydii* 1, 2, 4, 5, 8, 11, 14, and 15 cross-react with strains of *E. coli* O149, O87, O53, O79, O143, O105, O32, and O112, respectively [35]. In a study conducted by Liu et al., the presence of the *wzx* (flippase) and *wzy* (polymerase) genes in *E. coli* was identified, confirming the existence of a shared epitope similar to that found in the O antigen of the *S. boydii* 16 pentasaccharide unit. The presence of *S. boydii* 16-like somatic antigens in *E. coli* may reflect genetic similarities and possibly shared epitopes. While such antigenic convergence could influence bacterial properties, our study does not directly assess their biological or pathogenic consequences. This modification could significantly complicate the identification and classification of infections caused by these strains, necessitating the development of more specific diagnostic tools and approaches to detect and differentiate these infections from others caused by traditional *E. coli* pathotypes.

### 3.3. Phylogenetic Group Determination

Regarding identifying the phylogenetic group to which the *E. coli* strains isolated from irrigation water belonged, it was found that the A34 strain presented a B2 phylogenetic group; however, A51 did not achieve any group identification (Table 1). The B2 phylogenetic group of *E. coli* has diverse pathogenic potential and contains commensal but also includes intestinal and extraintestinal pathogenic isolates. The genetic profile of the B2 phylogenetic group of *E. coli* has been associated in the literature with horizontal gene transfer and accumulation of virulence traits. Although we observed virulence and resistance markers in our isolates, our study did not include genomic analyses to confirm such events [36]. The strains belonging to the B2 phylogenetic group of *E. coli* have been observed to have more virulence genes and genes related to antibiotic resistance than other phylogenetic groups of *E. coli*. It is thought that the acquisition of these genes could have resulted from horizontal gene transfer events. Assigning *E. coli* strains to a particular phylogroup provides insight into their ecological niche, lifestyle, and propensity to cause disease [37]. Understanding the phylogenetic distribution of *E. coli* strains, particularly the higher prevalence of virulence and antibiotic resistance genes in the B2 group, is essential for advancing both diagnostic methods and broader public health strategies. This knowledge enables the development of more precise tools for early detection, tailored interventions, and effective monitoring programs across healthcare, agriculture, and environmental settings.

The prevalence of *E. coli* phylogenetic group B2 in agricultural samples found in primary production environments, such as irrigation water, agricultural soils, and fresh produce, has been reported in previous studies. Corzo et al. [38] reported the presence of *E. coli* belonging to the phylogenetic group B2 in Northern Mexico in irrigation water used for tomato crops (2%) and in jalapeño pepper crop soil (11.1%). This is also consistent with the findings of Jonhson et al. [39], who reported that only 5% of *E. coli* strains isolated from surface waters from Minnesota and Wisconsin belonged to phylogroup B2. Wild animals can be carriers of *E. coli* strains, such as those characterized in a deer population in Mexico, in which it was found that 13.6% belonged to the phylogenetic group B2 [28]. Similarly, in China, *E. coli* B2 has been detected in 26.7% of farmed ducks from farms in the Zhanjiang area, mainly carrying the *bla*_TEM_ gene [40].

Phylogenetic analysis of *E. coli* strains isolated from irrigation water revealed the presence of group B2, known for its pathogenic potential and antibiotic resistance. These results underscore the importance of monitoring irrigation water quality and understanding human-animal-environment interactions in the spread and genetic diversity of *E. coli*, contributing to a better understanding of public health and food safety risks.

### 3.4. Virulence-Associated Genes and ERIC-PCR

Another essential feature is that the A51 strain of *E. coli* presented the *afa/draBC* genetic encoding for the Afa/DraBC adhesin (Table 3). Several virulence factors have been identified in the accessory genome of *E. coli,* encoding proteins involved in adhesion, invasion, motility, delivery of effector molecules, and toxicity. In the A51 strain of *E. coli*, the presence gene *afa/draBC* encodes the adhesin Afa/DraBC, which mainly allows the bacterium to attach to host cells. Specifically, Afa/DraBC makes it easier for *E. coli* to bind to cells in the urinary and gastrointestinal tract, allowing it to colonize and establish an infection in the host. In addition to its adhesive function, Afa/DraBC has been shown to play a role in phagocytosis resistance and survival in harsh environments. Not all strains of *E. coli* contain Afa/DraBC, but those that can be pathogenic and cause infections in humans and animals contain them [41]. This fimbrial adhesin also contributes to biofilm formation, a key factor in chronic and recurrent infections, particularly in urinary tract infections and gastrointestinal diseases. These mechanisms by which Afa/DraBC enhances bacterial survival and virulence could lead to the development of targeted therapeutic strategies or vaccines aimed at disrupting its function, offering the potential for more effective treatments against *E. coli*-induced infections.

The *afa/draBC* gene has been reported to be mobilizable via horizontal gene transfer in other *E. coli* strains [42]. While its presence in A51 suggests the possibility of such genetic events, our study does not provide direct evidence of the transfer mechanism involved. In addition, some studies suggest that *afa* genes can be transferred between different strains of *E. coli* via mobile DNA elements, such as plasmids or transposons [43].

Several studies have demonstrated the presence of the *afa/draBC* gene in *E. coli* strains. Such is the case reported by Mihailovskaya et al. [44], who reported the presence of this gene in 61.2% of isolates from healthy cows and calves in Perm Krai. The presence of the *afa/dra* gene has also been reported in chickens (1.25%) and swine (2.29%) farmed in South Korea [45]. Since wild animals can carry these strains and have been found in agricultural soil samples and fresh produce, it is plausible that they can also be transported into irrigation water through various pathways, such as fecal deposition of contaminated animals, runoff of contaminated water from agricultural areas, or direct deposition of bacteria in water. Therefore, *E. coli* strains containing the *afa/draBC* gene in irrigation water could pose a potential risk to public health if these strains survive and persist in that environment, mainly if used to irrigate crops intended for human consumption.

**Figure 2:**
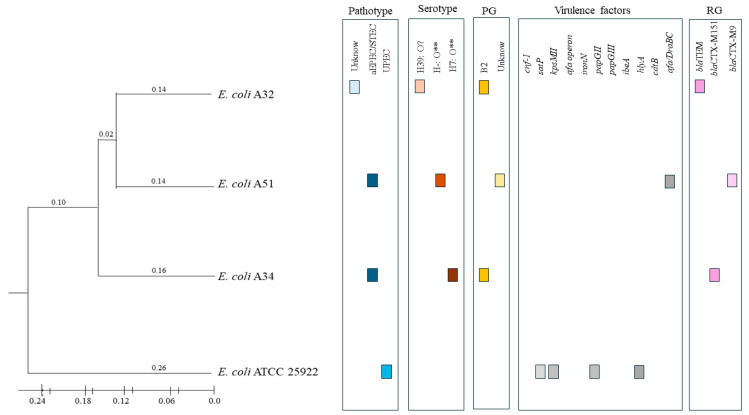
Dendogram of *E. coli* strains (A32, A34, A51, and ATCC 25922) was obtained by clustering ERIC profiles using the UPGMA algorithm and the DICE similarity coefficient. PG: Phylogenetic group; RG: Resistance genotype. ** indicates a somatic antigen (O) related to *Shigella boydii* 16.

In addition, to establish a phylogenetic relationship between *E. coli* strains A34 and A51, a phylogeny based on ERIC-PCR was performed (Figure 2). It was observed that the A32 and A51 strains presented a similarity of more than 80% according to their ERIC profiles. The A34 was also quite similar, but there was more variation in the ERIC profiles, so it is in another branch. According to the ERIC profile, *E. coli* ATCC 25922 is far removed from its isolates; this is interesting since 25922 is considered uropathogenic according to its virulence gene load. These results suggest genetic differences between the studied strains, which could affect their pathogenicity and virulence capacity.

### 3.5. Antibiotic Susceptibility and Extended-Spectrum Β-Lactamases Genes

*E. coli* strains A34 and A51 resisted various antibiotics, including cephalosporins, carbapenems, beta-lactams, quinolones, penicillins, and sulfonamides (Table 4). These strains carried *bla*_CTX-M-151_ (A34) and *bla*_CTX-M-9_ (A51), which are associated with extended-spectrum β-lactamase production and resistance to cephalosporins and other β-lactam drugs. Although these genes were detected by conventional PCR, no quantitative gene expression analysis or functional assays were performed. Therefore, while their presence suggests a potential role in resistance, further studies are needed to confirm their expression and enzymatic activity in these isolates. It is well known that *E. coli* develops antibiotic resistance through various mechanisms, including acquiring genes encoding specific enzymes [46]. This study observed that *E. coli* strains A34 and A51 did not have genes related to quinolone resistance. Therefore, this resistance could be due to other mechanisms, such as upregulation of efflux pumps or mutations in DNA gyrase or topoisomerase IV [47]. Among the significant mutations are those in the *gyrA*, *gyrB, parC,* and *parE* genes [48]. However, the most mutated residues in ciprofloxacin-resistant strains are serine and aspartic/glutamic acid in the *gyrA*/*parC* IV helix [49]. The identification of these mutations could provide important insights into the molecular basis of quinolone resistance, which could facilitate the development of novel diagnostic tools or therapies targeting these resistance mechanisms. Additionally, understanding how these mutations evolve and spread across bacterial populations is crucial for managing antibiotic resistance and preventing the emergence of multidrug-resistant strains.

The selection of antibiotics used in food production is geographically variable and is influenced by factors such as the production system, the type of agriculture, and the legislation in force. The indiscriminate use of antibiotics in agriculture and the release of these compounds into the environment through various sources, such as human waste and their application in agriculture, has resulted in a prolonged presence of antibiotics in the environment. This prolonged exposure has led to the emergence of resistant bacteria [50]. Bacteria that were initially susceptible to antibiotics have developed resistance, primarily through modification of their target binding sites, enzyme neutralization, or membrane permeability changes induced by efflux pumps. In addition, bacteria can acquire antibiotic-resistance genes from other bacteria or phages through horizontal gene transfer [51].

The use of antibiotics in agriculture raises concerns because of the risk of spreading resistance to clinically significant bacteria. Accordingly, the Food and Agriculture Organization of the United Nations has recommended improved awareness, capacity building, monitoring, and management of antimicrobial use in food and agriculture, as well as the promotion of good practices in food and agricultural systems and the prudent use of antimicrobials in its plan to combat antimicrobial resistance [52]. The environment serves as a reservoir of antibiotic resistance genes transferable to clinically significant pathogenic bacteria, providing a diversified gene pool [53].

Irrigation water represents a significant source of contamination of fresh produce with antibiotic-resistant bacteria. This phenomenon is rising due to the selective pressure exerted by anthropogenic factors [54]. Aquatic ecosystems have been identified as primary reservoirs of antibiotic-resistant bacteria, and determinants of resistance in these environments have been documented.

A study by Bolukaoto et al. [26] reported the presence of *E. coli* strains resistant to cefuroxime (100%), ceftazidime (86%), and cefotaxime (81%) in ambient water in Johannesburg, South Africa. Likewise, Montero et al. [55] identified the presence of extended-spectrum beta-lactamase (ESBL) producing *E. coli* in 58% of isolates obtained from irrigation water used to produce horticultural products in Ecuador. Similarly, Gekenidis et al. [54] demonstrated the presence of 22% *E. coli* ESBL in isolates collected from irrigation water in various horticultural areas in Switzerland. Agricultural irrigation water in Valencia, Spain, showed a high prevalence of multidrug-resistant *E. coli* strains (70.4%), and strains had the *bla*_TEM_ gene (96%). Likewise, these strains showed resistance to sulfonamides (93.3%), quinolones (73.3%), and tetracyclines (66.7%) [56]. This indicates that regular monitoring of irrigation water is required, including parameters related to antibiotic resistance. Similarly, multi-resistant strains of *E. coli* (17%) were detected in irrigation water of food products in Northwestern Mexico, which showed excellent resistance to cefotaxime (48.3%), ampicillin (44.8%), and tetracyclines (37.9%) [57]. This represents a potential source of human infection, so routine monitoring of irrigation water from food crops should be performed.

Regarding resistance isolated from farm animals, Mihailovskaya et al. [44] found that 32.7% of *E. coli* strains isolated from healthy cattle were shown to be multidrug-resistant to at least three groups of antibiotics. In addition, beta-lactam resistance genes were identified in significant proportions: *bla*_TEM_ (100%), *bla*_SHV_ (31.6%), and *bla*_CTX-M_ (26.3%). These findings underscore the urgency of addressing and monitoring antimicrobial resistance, as it poses risks to human and animal health, highlighting the importance of genetic characterization for effectively managing this problem. Several anthropogenic activities have been reported as sources of irrigation water pollution, including animal intrusions and the discharge of poultry and pig effluents [58].

The identification of hybrid *E. coli* strains harboring virulence and resistance genes in irrigation water supports the relevance of the One Health approach. Irrigation water represents a convergence point for environmental, agricultural, and human activity, facilitating the potential transmission of pathogenic and antimicrobial-resistant bacteria across ecosystems. These findings underscore the need for integrated monitoring systems that include environmental samples alongside clinical and veterinary surveillance. Addressing such microbial threats requires coordinated action across sectors to mitigate risks to public health, food safety, and ecological stability.

This study presents several limitations that should be acknowledged. First, the molecular characterization of the strains was limited to conventional PCR, without functional validation such as gene expression analysis or phenotypic assays for toxin production or antibiotic degradation. Second, the classification of hybrid pathotypes and inference of horizontal gene transfer were based on gene detection and phenotypic traits, without genomic confirmation through whole-genome sequencing, which would be necessary to assess gene synteny, mobility elements, and epitope-level comparisons, particularly in relation to the observed antigenic similarity with *Shigella boydii* 16. In addition, the use of ERIC-PCR provided only a low-resolution overview of phylogenetic relationships among the isolates. Lastly, the adhesion assay on HeLa cells was qualitatively described, lacking quantitative scoring or molecular insight into host-pathogen interaction mechanisms.

Future studies should address these limitations by incorporating whole-genome and transcriptomic approaches to validate the genetic context of virulence and resistance markers. Functional assays such as ELISA, reporter systems, or in vivo infection models (e.g., intestinal organoids or murine models) are also recommended to evaluate the pathogenic potential of the isolates. Moreover, expanding the number of strains studied from diverse agricultural sources would provide a more comprehensive understanding of environmental *E. coli* diversity and risk. These efforts will strengthen the evidence base for integrated microbial risk assessments in farming systems and align with the One Health approach to food safety and public health.

## 4. Conclusions

This study highlights the presence and characterization of heteropathogenic strains of *E. coli* (aEPEC/STEC) isolated from irrigation water. The A34 and A51 strains showed aggregative and diffuse adhesion patterns, with the A34 strain belonging to the phylogenetic group B2, known for its pathogenic potential and antibiotic resistance. Both strains shared somatic antigens with *S. boydii* 16, suggesting possible horizontal gene transfer events. In addition, these strains were resistant to multiple antibiotics, including cephalosporins and β-lactams, due to the presence of the *bla*_CTX-M-151_ and *bla*_CTX-M-9_ genes. Identifying antibiotic-resistant *E. coli* strains in irrigation water is a public health concern, especially in the context of contamination of fresh produce. The finding underscores the importance of implementing systematic water quality monitoring programs, particularly microbiological indicators and resistance profiles. Strengthening surveillance policies can help detect emerging strains early and reduce the risk of transmission through the food chain. Moreover, integrating Good Agricultural Practices, regular water treatment protocols, and education on the responsible use of antibiotics is critical to mitigating the dissemination of resistant bacteria. Ensuring the microbiological safety of water used in food production is essential to protect consumer health and maintain the integrity of the food supply system

## Figures and Tables

**Table 1:** Pathotypes, serotyping, and phylogenetic groups of *E. coli* strains.

Strain	Source	Pathotype	Serotyping	Phylogenetic Group
A34	Irrigation water	aEPEC/STEC	H7: O**	B2
A51	Irrigation water	aEPEC/STEC	H-: O**	Unknown

**: Somatic antigenic relation with *Shigella boydii* 16.

**Table 2:** Agglutination titers of unabsorbed and absorbed *E. coli* A34, A51, 64474, 0179, 0188, and *S. boydii* 16 sera.

Antigen	Titers of Unabsorbed Sera				Titers of Sera Absorbed with Boiled Cultures					
					*E. coli* 64474 absorbed with:			*S. boydii* 16 absorbed with:		
	*E. coli* 64474	*E. coli* 0179	*E. coli* 0188	*S. boydii* 16	*E. coli* 0179	*E. coli* 188	*S. boydii* 16	*E. coli* 64474	*E. coli* 0179	*E. coli* 0188
*E. coli* A34	1:100	-	-	1:800	-	-	-	-	1:800	1:1600
*E. coli* A51	1:100	-	-	1:800	-	-	-	-	1:800	1:1600

**Table 3:** Virulence-associated genes of the *E. coli* A34 and A51.

Strain	*cnf-1*	*satP*	*kpsMII*	*afa operon*	*iroN*	*afa/draBC*	*papGII*	*papGIII*	*ibeA*	*hlyA*	*cdtB*
A34	-	-	-	-	-	-	-	-	-	-	-
A51	-	-	-	-	-	+	-	-	-	-	-

**Table 4:** Antibiotic susceptibility and extended-spectrum β-lactamases genes of the *E. coli* A34 and A51.

Strain	Resistotype	ESBL Genes	ESBL Non-Genes
A34	CTX, CXM, CRO, FEP, MEM, ETP, AMC, AMP, CIP, STX	*bla*_CTX-M-151_	--
A51	CTX, CXM, CRO, FEP, MEM, ETP, AMC, AMP, CIP, SXT	*bla*_CTX-M-9_	--

Cefotaxime (CTX); cefuroxime (CXM); ceftriaxone (CRO); cefepime (FEP); meropenem (MEM); ertapenem (ETP); amoxicillin/clavulanic acid (AMC); ampicillin (AMP); ciprofloxacin (CIP); and sulfamethoxazole/trimethoprim (STX).

## Data Availability

The dataset could be available on request.

## Supplementary Materials

The following supporting information can be downloaded at: https://www.mdpi.com/article/10.3390/pathogens14060549/s1, Table S1: Specific oligonucleotides were used in this study. References [10,17,18,22,59,60,61,62,63,64,65,66,67,68,69,70,71,72,73,74] are cited in the supplementary materials.
