# Supplementary material for: Virulence and Antibiotic Resistance of aEPEC/STEC Escherichia coli Pathotypes with Serotype Links to Shigella boydii 16 Isolated from Irrigation Water
Source: Pathogens. 2025 Jun 1;14(6):549. doi: 10.3390/pathogens14060549 (PMC12195767; doi:10.3390/pathogens14060549)
Supplement: Supplementary file 1 [file pathogens-14-00549-s001.zip › pathogens-3607328-supplementary.pdf]

1 **Supplementary materials**

2 **Table S1.** Specific oligonucleotides were used in this study.

Gene	Sequence (5'- 3')	Amplicon size	Reference
ybbw	F:tgattggcaaaatctggccg	667 bp	[18, 17]
	R:atactggcaatcagtacgcc		
eaeA	F:caggtcgtcgtgtctgctaaa	570 bp	[59]
	R:tcagcgtggttggatcaacct		
bfpA	F:aatggtgcttgcgcttgctgc	326 bp	[60]
	R:gccgctttatccaacctggta		
stx1	F:tttacgatagacttctcgac	227 bp	[61]
	R:cacatataaattatttcgctc		
stx2	F:cccagtcacgacgttgta	460 bp	[10]
	R:tatactatcgtgcctttcca		
LT	F:ggcgacagattataccgtgc	750 bp	[62]
	R:ccgaattctgttatatatgtc		
ST	F: atttttctttctgtattgtctt	190 bp	[63]
	R: cacccggtacaagcaggatt		
daaE	F: tgactgtgaccgaagagtgc	380 bp	[64]
	R: ttagttcgtccagtaaccccc		
iaI	F: ctggatggtatggtgagg	320 bp	[10]
	R: ggaggccaacaattatttcc		
EAEC	F:ctggcgaaagactgtatcat	630 bp	[63]
plasmid	R:caatgtatagaaatccgctgtt		
chuA	F: atggtaccggacgaaccaac	288 bp	[65]
	R: tgccgccagtaccaaagaca		
yjaA	F: caaacgtgaagtgtcaggag	211 pb	[65]
	R: aatgcgttcctcaacctgtg		
TspE4. C2	F: cactattcgtaaggtcatcc	152 bp	[65]
	R: agtttatcgctgcgggtcgc		
arpA	F: aacgctattcgccagcttgc	400 bp	[65]
	R: tctccccataccgtacgcta		
trpA	F: cggcgataaagacatcttcac	489 bp	[65]
	R: gcaacgcggcctggcggaag		
cnf-1	F: ctcgcccagtgattaggtattc	3,100 bp	[66]
	R: gcgctaacaaaacagcacaagg		
satP	F: agcaagctgttagtaaccaacc	880 bp	[66]
	R: gagccgctgtctccgaata		
kpsMII	F:gatacgccaacagggaaatg	272 bp	[67]
	R:catccagacgataagcatgagca		
afa operon	F:gagtcacggcagtcgcggcgg	207 bp	[68]
	R:ttcaccggcgaccacggatctcc		
iroN	F:aagtcaaagcaggggttgcccg	667 bp	[67]
	R:gacgccgacattaagacgcag		
afa/draBC	F:ggcagagggccggcaacaggc	559 bp	[69]
	R:cccgtaacgcgccagcatctc		

papGII	F: ggaatgtggtgattactcaaagg<br>R: tccagagactgtgcagaaggac	562 bp	[70]	3
papGIII	F:catggctggttgttcctaaacat<br>R: tccagagactgtgcagaaggac	421 bp	[71]	4
ibeA	F:aggcaggtgtgcgccgcgtac<br>R:tggtgctccggcaaaccatgc	170 bp	[69]	
hlyA	F:actcatgttggtaaagtatcagaat<br>R: agccagtacagtgcttatcgttg	1,280 bp	[66]	
cdtB	F:tatgatagcctctttatcgtcgtcgtctg<br>R:agaggagagttagagcctaatgatagcctct	805 bp	[70]	
CTX-M1&8	F:tgtgcagyaccagtaargykatg<br>R:tarrtsaccagaayvagcggc	583 bp	[71]	
CTX-M-2	F:cgagtggcagtaccagtaagg<br>R:cgatatcgttggtggtgc	540 bp	[71]	
CTX-M-9	F:atggtgacaaagagagtgcaa<br>R:aatatcattggtggtgccgtag	747 bp	[71]	
CTX-M-151	F:gcggccatgataggtacg<br>R:aaagtaagtcacaataaccagcg	786 bp	[71]	
TEM	F:caacattttcgtgtcgccc<br>R:gcttaatcagtgaggcacc	844 bp	[71]	
SHV	F:tattatctccctgttagcca<br>R:cgctctgctttgttattc	783 bp	[71]	
qepA	F:gcaggtccagcagcggggtag<br>R:ggacatctacggcttcttcg	617 bp	[72, 73]	
aac(6´)-lb-cr	F: ttgcgatgctctatgagtggcta<br>R:ctcgaatgcctggcgtgttt	482 bp	[74]	
ERIC-PCR	ERIC-1 atgtaagctcctggggattcac<br>ERIC-2 aagtaagtgactggggtgagcg		[22]	
